# Pharmacological Analysis of the Activation and Receptor Properties of the Tonic GABA_C_R Current in Retinal Bipolar Cell Terminals

**DOI:** 10.1371/journal.pone.0024892

**Published:** 2011-09-15

**Authors:** Stefanie M. Jones, Mary J. Palmer

**Affiliations:** Neuroscience Group, Institute for Science and Technology in Medicine, Keele University, Keele, United Kingdom; Dalhousie University, Canada

## Abstract

GABAergic inhibition in the central nervous system (CNS) can occur via rapid, transient postsynaptic currents and via a tonic increase in membrane conductance, mediated by synaptic and extrasynaptic GABA_A_ receptors (GABA_A_Rs) respectively. Retinal bipolar cells (BCs) exhibit a tonic current mediated by GABA_C_Rs in their axon terminal, in addition to synaptic GABA_A_R and GABA_C_R currents, which strongly regulate BC output. The tonic GABA_C_R current in BC terminals (BCTs) is not dependent on vesicular GABA release, but properties such as the alternative source of GABA and the identity of the GABA_C_Rs remain unknown. Following a recent report that tonic GABA release from cerebellar glial cells is mediated by Bestrophin 1 anion channels, we have investigated their role in non-vesicular GABA release in the retina. Using patch-clamp recordings from BCTs in goldfish retinal slices, we find that the tonic GABA_C_R current is not reduced by the anion channel inhibitors NPPB or flufenamic acid but is reduced by DIDS, which decreases the tonic current without directly affecting GABA_C_Rs. All three drugs also exhibit non-specific effects including inhibition of GABA transporters. GABA_C_R ρ subunits can form homomeric and heteromeric receptors that differ in their properties, but BC GABA_C_Rs are thought to be ρ1-ρ2 heteromers. To investigate whether GABA_C_Rs mediating tonic and synaptic currents may differ in their subunit composition, as is the case for GABA_A_Rs, we have examined the effects of two antagonists that show partial ρ subunit selectivity: picrotoxin and cyclothiazide. Tonic and synaptic GABA_C_R currents were differentially affected by both drugs, suggesting that a population of homomeric ρ1 receptors contributes to the tonic current. These results extend our understanding of the multiple forms of GABAergic inhibition that exist in the CNS and contribute to visual signal processing in the retina.

## Introduction

GABA, the major inhibitory neurotransmitter in the CNS, evokes transient postsynaptic currents (IPSCs) via ionotropic GABA_A_ and GABA_C_ receptors, as well as slower synaptic responses via metabotropic GABA_B_ receptors (GABARs). In addition, there is increasing evidence that GABA evokes a tonic increase in membrane conductance by activating extrasynaptic GABA receptors, either as a result of spill-over from synapses or via a non-synaptic mechanism [1]. Tonic GABAR currents are mediated by GABA_A_Rs in brain regions such as the hippocampus, cerebellum and thalamus, where they have a role in controlling neuronal excitability and network interactions [2], [3]. In the retina, a GABA_C_R-mediated tonic current occurs in the synaptic terminals of bipolar cells (BCs), which similarly regulates membrane excitability [4], [5]. Bipolar cell terminals (BCTs) also exhibit rapid synaptic GABA_A_R and GABA_C_R currents that mediate feedback inhibition and limit BC glutamate release, thereby modulating the light responses of ganglion cells, the output cells of the retina [6].

We have found that the tonic GABA_C_R current in BCTs, like some tonic GABA_A_R currents [7]–[10], is not dependent on vesicular GABA release [11]. The alternative source of GABA is currently unknown but does not appear to involve reversal of GABA transporters or release via hemichannels or P2X_7_ receptors [11]. It was recently shown that the tonic release of GABA from cerebellar glial cells can occur via Bestrophin 1 (Best1) Cl^-^ channels [12], which have a significant permeability to large anions such as thiocyanate, gluconate and glutamate [13], [14]. In addition, volume-regulated anion channels (VRACs) have been implicated in the non-vesicular release of neurotransmitters [15]. Astrocytic or neuronal release via anion channels may therefore be a potential source of GABA for activating the tonic GABA_C_R current in BCTs.

Tonic GABA_A_R currents are mediated by receptors that differ in their subunit composition from synaptic GABA_A_Rs, conferring distinct receptor properties that are suited to their localization and function, such as high GABA sensitivity and reduced desensitization [16], [17]. GABA_C_Rs are composed of ρ subunits which are highly expressed in the retina but are also localized to various brain regions including the midbrain, thalamus, hippocampus and cerebellum [18]. BC GABA_C_Rs are believed to be ρ1-ρ2 heteromers, although ρ subunits can also co-assemble with GABA_A_R γ subunits [19], [20]. Heterologous expression of ρ1 and/or ρ2 subunits reveals differences in receptor properties, for example ρ1 homomers exhibit higher GABA sensitivity, lower conductance and slower deactivation than ρ2 homomers, with heteromeric ρ1-ρ2 receptors generally showing intermediate properties [21]–[24]. However, it is unknown whether receptor subunit diversity contributes to the different forms of GABA_C_R-mediated inhibition in BCTs.

To further investigate the activation and receptor properties of GABA_C_Rs mediating the tonic current in BCTs, we have examined the effect of anion channel inhibitors and subunit-selective antagonists on spontaneous and evoked GABA_C_R currents recorded directly from BCTs in goldfish retinal slices. We find evidence for a role of DIDS-sensitive anion channels/exchangers in tonic GABA release, and for a contribution of homomeric ρ1 receptors to the tonic GABA_C_R current.

## Methods

Goldfish (*Carassius auratus*) were maintained in a 12 hour dark/light cycle at 16°C. Prior to use, light-adapted goldfish were dark-adapted for 1 hour to facilitate removal of the pigment epithelium. Goldfish were killed by decapitation followed immediately by destruction of the brain and spinal cord under Schedule 1 of the UK Animals (Scientific Procedures) Act 1986. The experiments were approved by Keele University's Central Animal Facility Management Committee. The eyeballs were removed and retinae dissected out and treated for 25 minutes with hyaluronidase to remove vitreous humor. Each retina was quartered, placed ganglion cell layer down on filter paper and kept until needed at 4°C in medium comprising (mM): NaCl (127), KCl (2.5), MgCl_2_ (1.0), CaCl_2_ (0.5), Hepes (5), glucose (12), adjusted to pH 7.45 with NaOH. Slices were cut at 250 µm intervals, transferred to the recording chamber and perfused (1 ml.min^-1^) with medium comprising (mM): NaCl (108), KCl (2.5), MgCl_2_ (1.0), CaCl_2_ (2.5), NaHCO_3_ (24), glucose (12), gassed with 95% O_2_/5% CO_2_, pH 7.4. For Ca^2+^-free extracellular solution, CaCl_2_ was omitted and MgCl_2_ was increased to 3.5 mM, to maintain the divalent cation concentration. The osmolarities of the 2.5 mM Ca^2+^ and Ca^2+^-free extracellular solutions were 267 mOsm and 269 mOsm respectively. Slice preparation and recordings were performed at room temperature (18–22°C), in daylight conditions.

Drugs were bath-applied via the extracellular solution and locally-applied via pressure application from a low resistance glass micropipette (∼5 µm tip diameter) positioned 25-50 µm from the recorded BCT using a Picospritzer II (Intracell, Royston, UK). GABA and L-glutamate solutions for local application also contained bicuculline (50 µM). L-glutamate-evoked GABA responses were evoked at intervals of at least 30 s in order to avoid short-term depression of GABA release [25]. Salts and drugs, including GABA, L-glutamate, 5-nitro-2-(3-phenylpropylamino)benzoic acid (NPPB), 2-(3-trifluoromethylphenylamino)-benzoic acid (flufenamic acid), 4,4′-diisothiocyanostilbene-2,2′-disulphonic acid (DIDS), picrotoxin, (1,2,5,6-tetrahydropyridin-4-yl)methylphosphinic acid (TPMPA), bicuculline, 1,2,5,6-tetrahydro-1-2-(diphenylmethylene)aminooxyethyl-3-pyridinecarboxylic acid hydrochloride (NO-711) and cyclothiazide were obtained from Tocris (Bristol, UK), Sigma-Aldrich (Gillingham, UK) and Fisher Scientific (Loughborough, UK).

Whole-cell voltage-clamp recordings were obtained from large Mb-type BC terminals, as described previously [26]. Most recordings were made from axon-severed terminals (determined by their capacitive current) [26] to eliminate currents arising from somatodendritic receptors and reduce the leak current. However, no differences in GABAR currents were observed between axon-severed terminals and the terminals of intact BCs. Patch pipettes (5–8 MΩ) were pulled from borosilicate glass and filled with solution comprising (mM): CsCl (115), Hepes (25), TEA-Cl (10), Mg-ATP (3), Na-GTP (0.5), EGTA (0.5), pH 7.2, 270 mOsm. CsCl-based intracellular solution was used to increase the driving force through GABARs at a holding potential of −60 mV.

Membrane current (I_M_) was recorded via an EPC-10 patch-clamp amplifier controlled by Patchmaster software (HEKA, Lambrecht/Pfalz, Germany). Series resistance (R_S_) was monitored and recordings were not used if I_M_ changes were accompanied by changes in R_S_. Off-line analysis was performed using IgorPro software (WaveMetrics, Lake Oswego, OR). Pooled data are expressed as mean ± SEM; statistical significance was assessed using paired or unpaired Student's *t* tests as appropriate, with P<0.05 considered significant.

## Results

### The role of anion channels: Effects of NPPB and flufenamic acid

In order to investigate the role of Best1 and other anion channels in non-vesicular GABA release in the retina, we tested the effect of anion channel inhibitors on GABA_C_R-mediated currents in BCTs. Recordings were made with CsCl-based intracellular solution at a holding potential of −60 mV, in the presence of bicuculline (50 µM) to block GABA_A_R-mediated spontaneous IPSCs (sIPSCs). The anion channel inhibitors were tested under both normal (2.5 mM) Ca^2+^ and Ca^2+^-free extracellular conditions; when no differences were observed between these conditions, the data has been pooled.

**Figure 1:**
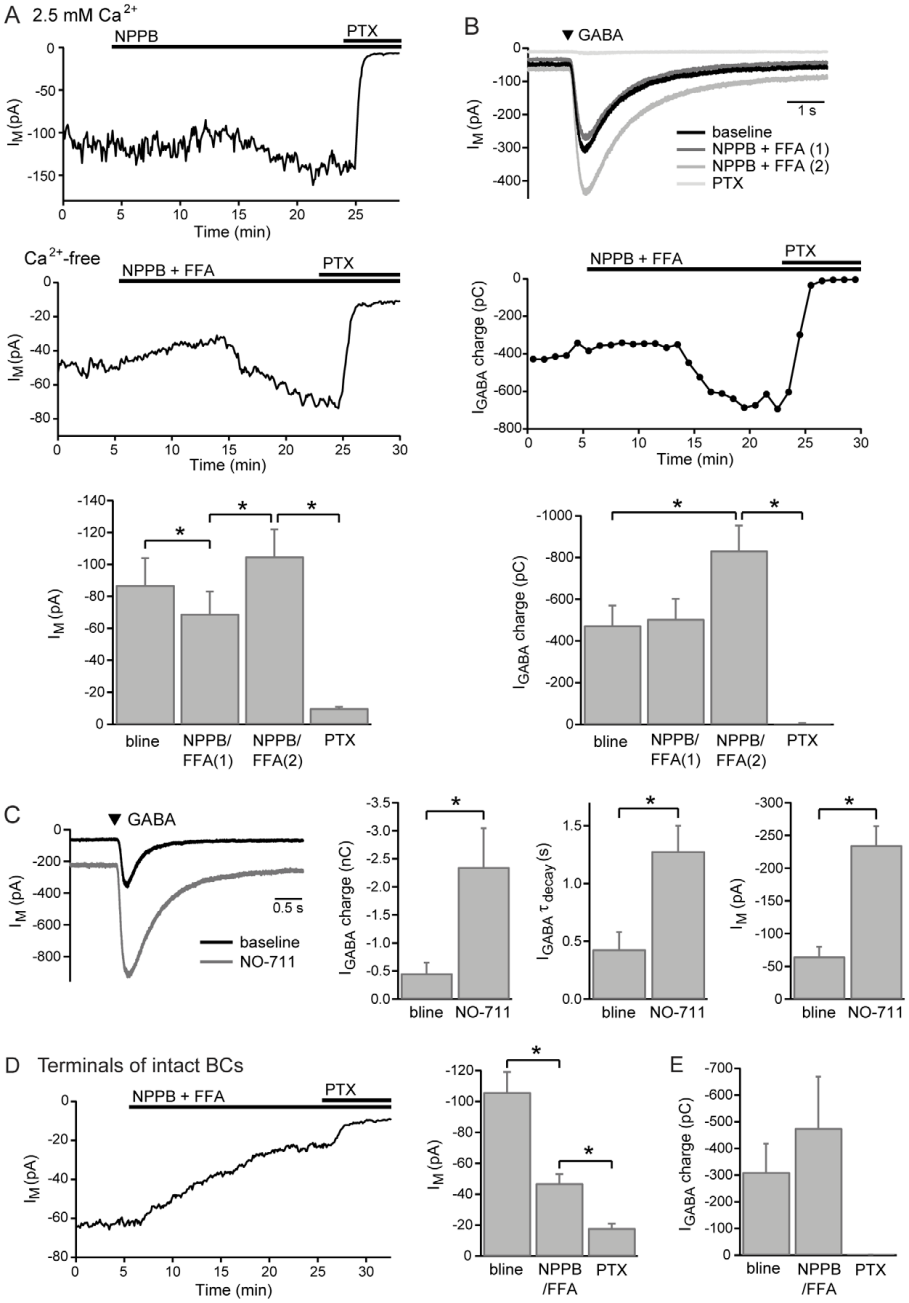
The effect of NPPB and FFA on GABA_C_R currents. A, Example experiments and mean data (2.5 mM Ca^2+^ n = 4, Ca^2+^-free n = 4) showing the effects of NPPB (50–100 µM) and FFA (100–200 µM) on the holding current in recordings from axon-severed BCTs, with subsequent addition of picrotoxin (PTX; 200 µM). NPPB/FFA(1) was measured 5–10 mins after drug application; NPPB/FFA(2) was measured 10–20 mins after application. B, Example GABA_C_R responses evoked by local application of GABA (100 µM, 100 ms) and the charge of GABA-evoked responses against time for the recording in Ca^2+^-free conditions in A, with mean data (2.5 mM Ca^2+^ n = 2, Ca^2+^-free n = 4) showing the effect of NPPB and/or FFA on the charge of GABA-evoked responses. C, Example responses and mean data (n = 6) showing the effect of the GAT-1 inhibitor NO-711 (3 µM) on GABA-evoked responses (100 µM, 50–100 ms), and the associated increase in the tonic GABA_C_R current. D, An example experiment in Ca^2+^-free extracellular solution and mean data (2.5 mM Ca^2+^ n = 3, Ca^2+^-free n = 4) showing the effect of NPPB (50 µM) and/or FFA (100–200 µM) on the holding current in recordings from the terminals of intact BCs, with subsequent addition of picrotoxin (200 µM). E, Mean data showing the effect of NPPB (50 µM) and/or FFA (100-200 µM) on the charge of GABA-evoked responses (2.5 mM Ca^2+^ n = 2, Ca^2+^-free n = 3) in recordings from the terminals of intact BCs. All experiments in this and subsequent figures were performed with CsCl-based intracellular solution at a holding potential of -60 mV in the presence of bicuculline (50 µM), unless stated otherwise. Example evoked currents show the average of 2–5 responses in each condition. Error bars represent SEM; * denotes P<0.05.

Application of the anion channel inhibitor NPPB (50-100 µM) to axon-severed BCTs initially evoked a small decrease, followed by an increase, in the holding current over the course of about 20 minutes (2.5 mM Ca^2+^ n = 2, Ca^2+^-free n = 2; fig. 1A). Application of flufenamic acid (FFA; 100–200 µM), either alone (2.5 mM Ca^2+^ n = 2) or in combination with NPPB (Ca^2+^-free n = 2), evoked the same biphasic effect (fig. 1A). The potentiated current in NPPB and/or FFA was subsequently inhibited by the addition of the GABAR antagonist picrotoxin (200 µM; 2.5 mM Ca^2+^ NPPB n = 1, FFA n = 1; Ca^2+^-free NPPB n = 1, NPPB+FFA n = 2; fig. 1A), confirming that it was mediated by GABA_C_Rs. Responses to locally-applied GABA (100 µM, 50–100 ms application) were monitored in the same experiments to check for direct inhibition of GABA_C_Rs by the anion channel blockers. The charge of GABA-evoked responses was not reduced by NPPB (n = 3), FFA (n = 1) or combined application (n = 2). Instead, a significant potentiation of GABA-evoked responses was observed, which occurred in parallel with the tonic current increase (fig. 1B). The GABA-evoked responses were subsequently fully blocked by picrotoxin (n = 4; fig. 1B).

The potentiating effects of NPPB and FFA on both the tonic current and exogenous GABA responses may result from inhibition of GABA uptake, as inhibition of GAT-1 by NO-711 (3 µM) exerts a similar, though more pronounced, potentiating effect on the tonic current [4] and on the charge of GABA-evoked responses (n = 6; fig. 1C). FFA and the related compound niflumic acid have previously been found to inhibit certain GAT isoforms to variable extents [27]. The small initial decrease in the holding current may indicate a minor contribution of NPPB/FFA-sensitive anion channels to non-vesicular GABA release, or may result from a non-specific effect of these drugs on other ion channels (see below).

A markedly different effect of NPPB and FFA was observed in recordings made from the terminals of intact BCs. Application of NPPB (50 µM; n = 3), FFA (100–200 µM; n = 2) or both in combination (n = 2) resulted in a significant reduction in the holding current (2.5 mM Ca^2+^ n = 3, Ca^2+^-free n = 4), which was subsequently further reduced by application of picrotoxin (200 µM; fig. 1D). Conversely, responses evoked by local application of GABA (100 µM, 50–100 ms) were potentiated by NPPB and/or FFA in 4 out of 5 of these recordings (fig. 1E). The inhibitory effect of NPPB and FFA on the holding current of intact BC recordings is most likely due to the additional action of these drugs as hemichannel inhibitors [28]-[31], as Mb-type BCs in goldfish retina are connected via gap junctions in their dendrites [32]. The hemichannels appear to account for a major part of the increased membrane conductance of intact BCs compared with axon-severed BCTs [26].

### The role of anion channels: Effects of DIDS

**Figure 2:**
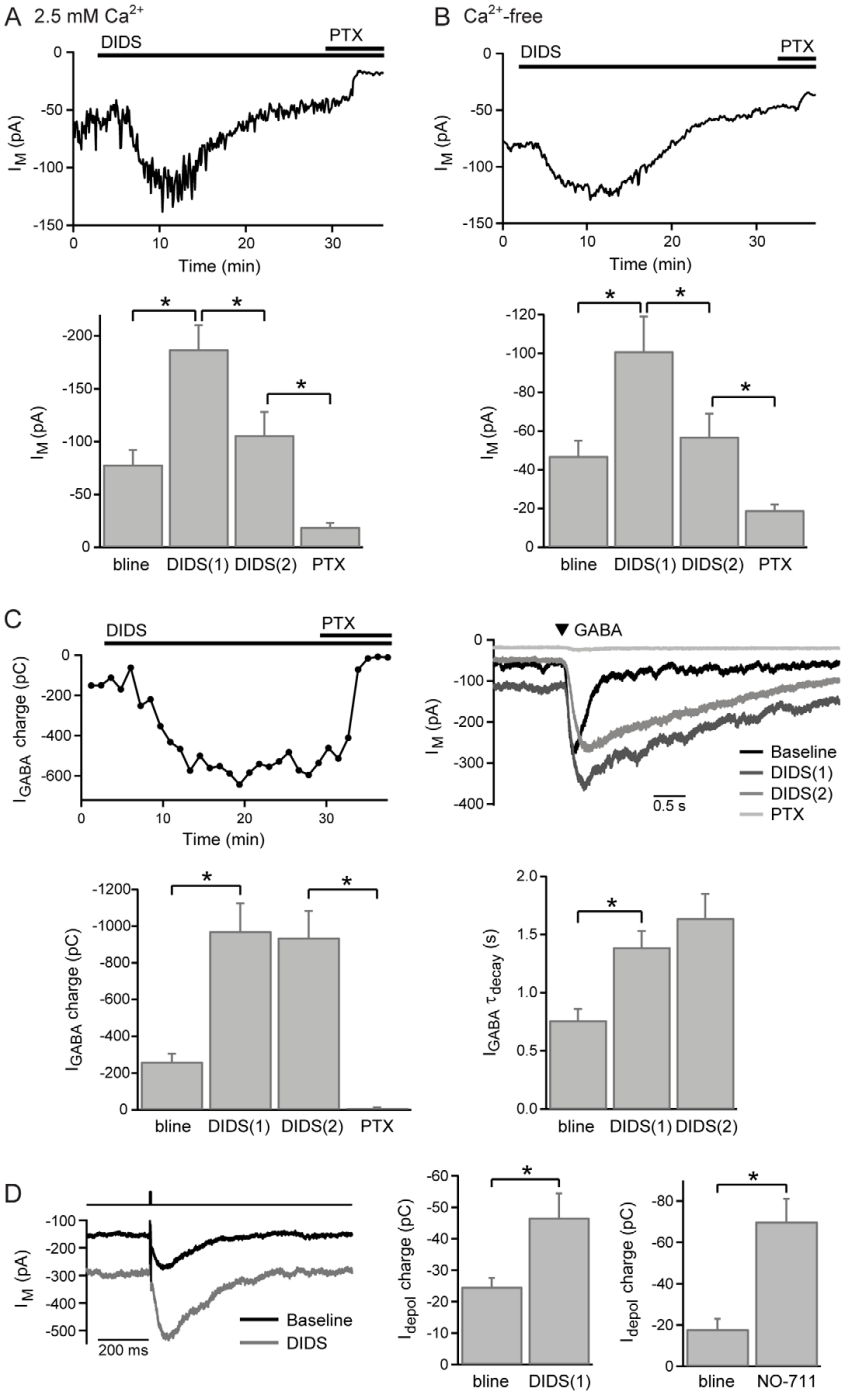
The effect of DIDS on GABA_C_R currents. A, Example experiment and mean data (n = 9) showing the biphasic effect of DIDS (500 µM) on the holding current in normal Ca^2+^ extracellular solution, with subsequent application of picrotoxin (200 µM). DIDS(1) was measured at the peak of the tonic current potentiation, DIDS(2) was measured following 15-30 mins of DIDS application, just prior to addition of picrotoxin. B, Example experiment and mean data (n = 11) showing a similar effect of DIDS (500 µM) in Ca^2+^-free extracellular solution. C, The charge of GABA-evoked responses (100 µM, 100 ms) against time and example responses for the experiment in A, with mean data (2.5 mM Ca^2+^ n = 7, Ca^2+^-free n = 7) showing the effect of DIDS on the charge and the decay time-constant of GABA-evoked responses. D, Example responses and mean data showing the effects of DIDS (500 µM; n = 5) and NO-711 (3 µM; n = 6) on the charge of GABA_C_R-mediated synaptic feedback responses evoked by brief BCT depolarization (to -10 mV for 5 ms).

The anion channel/exchanger inhibitor DIDS, which has no reported effect on hemichannels [28], reduces the tonic release of glutamate in hippocampal slices [33]. The effect of DIDS on the GABA_C_R tonic current in BCTs was therefore investigated. Application of DIDS (500 µM) to axon-severed BCTs in the presence of bicuculline (50 µM) initially caused a significant increase in the holding current (2.5 mM Ca^2+^ n = 9; Ca^2+^-free n = 11; fig. 2A,B), that was accompanied by a large increase in the amplitude and slowing of the decay of responses evoked by exogenous GABA (100 µM, 50–100 ms; n = 14; fig. 2C). In addition, DIDS potentiated GABA_C_R-mediated feedback currents evoked by activation of amacrine cell reciprocal synapses by brief BCT depolarization (to −10 mV for 5 ms; n = 5; fig. 2D). A similar, though larger, potentiation of synaptic feedback currents is evoked by the GAT-1 inhibitor NO-711 (3 µM; n = 6; fig. 2D) [4]. These effects are consistent with the reported action of DIDS as an inhibitor of GAT-1 [34].

However, in the continuing presence of DIDS, the tonic current gradually decreased. In 2.5 mM extracellular Ca^2+^, the holding current decreased from a peak of -187±23 pA to -106±22 pA following 15–30 minutes of DIDS application (n = 9), and was further reduced to −19±4 pA by subsequently addition of picrotoxin (200 µM; n = 7; fig. 2A). In Ca^2+^-free extracellular solution, the holding current decreased from a peak of −101±18 pA to -57±12 pA following 15–30 minutes of DIDS application (n = 11), and was further reduced to -19±3 pA by subsequently addition of picrotoxin (200 µM; n = 8; fig. 2B). During the period of tonic current reduction there was no significant change in the charge or rate of decay of responses evoked by exogenous GABA (2.5 mM Ca^2+^ n = 7, Ca^2+^-free n = 7; fig. 2C), which were subsequently eliminated by picrotoxin (200 µM; fig. 2C). These results strongly suggest that DIDS reduces the tonic current without directly affecting BCT GABA_C_Rs, and is therefore likely to be an inhibitor of the non-vesicular GABA release mechanism.

**Figure 3:**
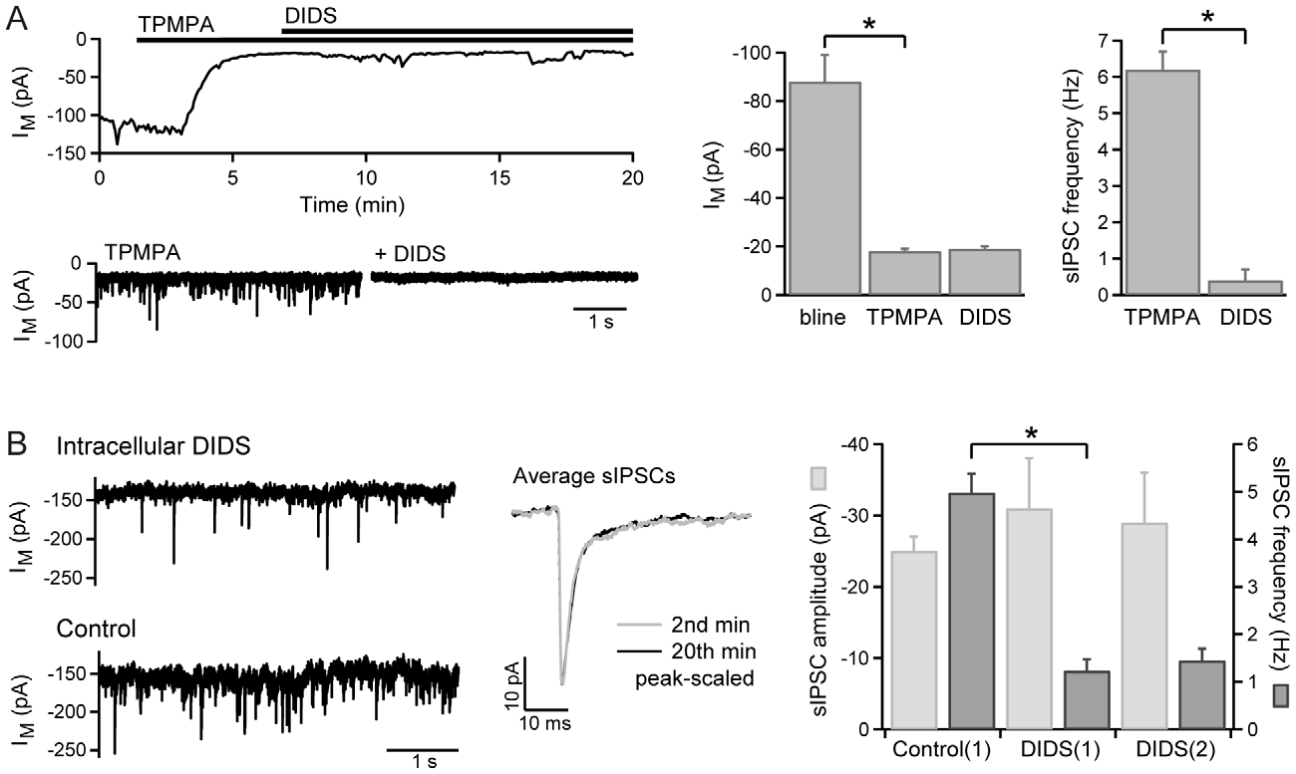
The effect of DIDS on GABA_A_R currents. A, An example recording and mean data (n = 4) showing that application of DIDS (500 µM) in the presence of TPMPA (200 µM) but not bicuculline has no effect on the holding current but inhibits spontaneous GABA_A_R-mediated IPSCs (sIPSCs). B, Example current traces from recordings with and without DIDS (500 µM) included in the intracellular solution, with average sIPSCs from a different recording with intracellular DIDS (500 µM), and mean sIPSC amplitude and frequency data in control recordings (n = 5) and recordings with intracellular DIDS (0.5–1 mM; n = 5). Control(1) and DIDS(1) were measured during the 2^nd^ minute after gaining whole-cell access, DIDS(2) was measured during the 6^th^ minute.

To confirm that the effects of DIDS are mediated by changes in the activation of GABA_C_Rs, and to investigate reported effects of DIDS as an inhibitor of GABA_A_Rs [35], DIDS (500 µM) was applied following inhibition of GABA_C_Rs with TPMPA (100–200 µM), in 2.5 mM Ca^2+^ extracellular solution without bicuculline. Under these conditions, GABA_A_R-mediated sIPSCs are observed [5]. In the presence of TPMPA, DIDS had no effect on the holding current, but did significantly reduce the frequency of sIPSCs (fig. 3A). DIDS has previously been used in combination with CsF as an intracellular inhibitor of GABA_A_Rs [36]–[38], based on evidence that it blocks other types of Cl^-^ channel from either side of the membrane [39], [40]. To ascertain whether DIDS acts as an intracellular blocker of GABA_A_Rs in BCTs, recordings were made with DIDS (0.5–1 mM) included in the intracellular solution. Intracellular DIDS had no effect on the amplitude of sIPSCs but significantly reduced their frequency, compared with control recordings (n = 5 for DIDS and control, sIPSCs measured during the 2^nd^ minute after gaining whole-cell access; fig. 3B). Intracellular DIDS appeared to reduce the longevity of whole-cell recordings (average duration 10±3 minutes, n = 5), but the amplitude and frequency of sIPSCs did not change during the course of recordings (2^nd^ minute compared with 6^th^ minute, n = 5; fig. 3B), and sIPSCs were still observed in the 20^th^ minute of the longest duration recording (fig. 3B). DIDS therefore appears to act as an intracellular inhibitor of GABA_A_Rs, but is more effective when applied extracellularly.

### GABA_C_R subunit composition: Picrotoxin-sensitivity

To investigate whether the ρ subunit composition of GABA_C_Rs mediating the tonic current differs from that of GABA_C_Rs mediating the relatively fast synaptic currents in BCTs, we have examined the effect of receptor antagonists that display some subunit-selectivity. As above, GABA_C_R currents were recorded at a holding potential of −60 mV with CsCl-based intracellular solution in the presence of the GABA_A_R antagonist bicuculline (50 µM).

**Figure 4:**
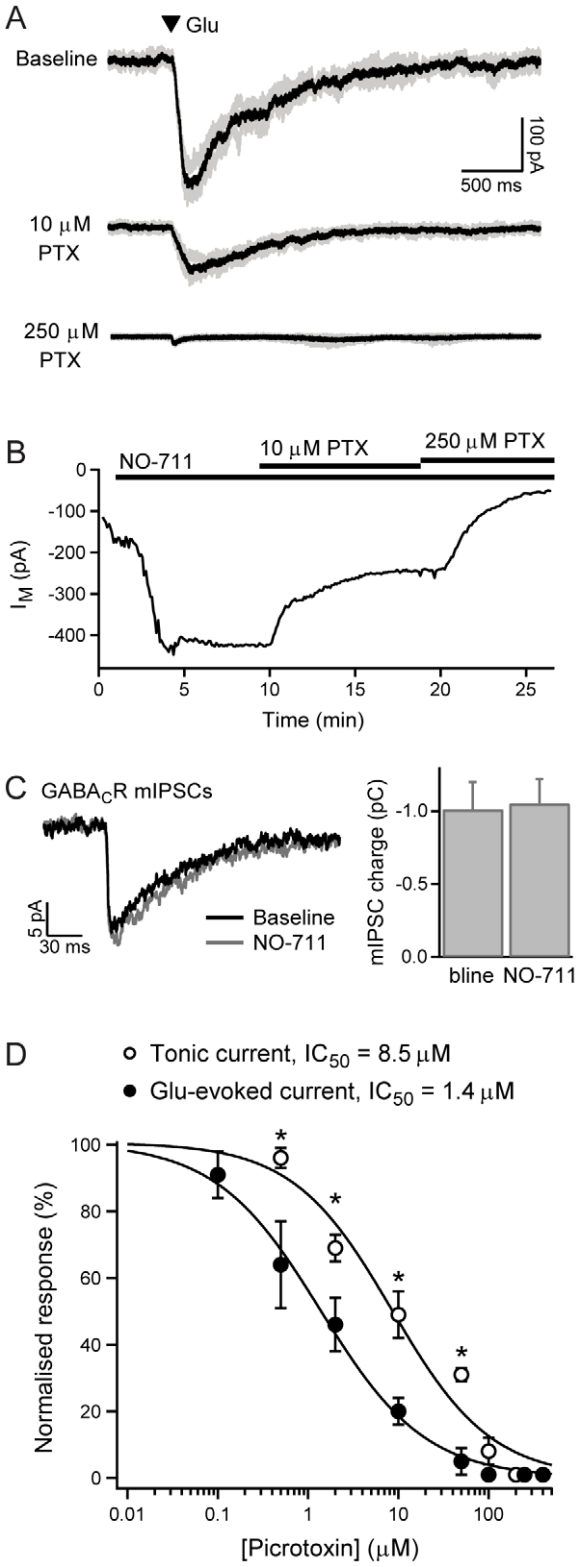
Picrotoxin-sensitivity of GABA_C_R currents. A, Example GABA_C_R responses evoked by local application of L-glutamate (glu; 100 µM, 10 ms) to activate reciprocal amacrine cell synapses, and their inhibition by picrotoxin. Three individual responses (grey) and the mean response (black) are shown for each condition. B, An example experiment showing inhibition of the tonic GABA_C_R current by the same concentrations of picrotoxin, following potentiation of the current by NO-711 (3 µM). C, Example average GABA_C_R-mediated mIPSCs during baseline and following application of NO-711 (3 µM), with mean data for average mIPSC charge under these conditions (n = 4). GABA_C_R mIPSCs were recorded in Ca^2+^-free extracellular solution to facilitate their detection, in the presence of bicuculline. D, Dose-response curves for picrotoxin inhibition of glu-evoked (n = 4–6) and tonic (n = 3–6) GABA_C_R currents, fit with Hill equations to give IC_50_ values.

The inhibition of GABA_C_Rs by picrotoxin is dependent on subunit composition, with homomeric ρ1 receptors exhibiting approximately 10-fold higher IC_50_ values than either ρ2 homomers or ρ1- ρ2 heteromers in the presence of high GABA concentrations [21]–[23], [41]. The picrotoxin-sensitivity of tonic and synaptic GABA_C_R currents in BCTs was therefore examined. Reciprocal amacrine cell synapses were activated by local application of L-glutamate (glu; 100 µM, 10 ms), which evokes large GABA_C_R-mediated currents in BCTs [11]. The glu-evoked responses were maximally-inhibited by 250 µM picrotoxin; application of 400 µM picrotoxin had no further effect (n = 3). After obtaining baseline glu-evoked responses, picrotoxin was applied at a concentration of 0.1, 0.5, 2, 10, 50 or 100 µM, followed by a concentration of 250 µM (n = 4–6 for each concentration; fig. 4A). The charge of the GABA_C_R-mediated component of glu-evoked responses was normalized to the size of the baseline GABA_C_R response and plotted versus picrotoxin concentration (fig. 4D). A fit of the dose-response plot with a Hill equation gave an IC_50_ value of 1.4 µM.

To determine the picrotoxin-sensitivity of the tonic GABA_C_R current, it was first potentiated by application of NO-711 (3 µM) [4]. NO-711 appears to exert its effects solely via inhibition of GABA uptake rather than via any direct action on GABA_C_Rs, as GABA_C_R-mediated mIPSCs [5] are not affected by application of NO-711 (fig. 4C). Following the establishment of a stable baseline tonic current in NO-711, picrotoxin was applied at a concentration of 0.5, 2, 10, 50, 100 or 200 µM, followed by a maximal concentration of 250 µM (n = 3-6 for each concentration; fig. 4B). The amplitude of the GABA_C_R-mediated tonic current was normalized to the baseline current and plotted versus picrotoxin concentration (fig. 4D). A fit of the dose-response plot with a Hill equation gave an IC_50_ value of 8.5 µM. The amount of inhibition of the tonic GABA_C_R current was statistically different from that of glu-evoked GABA_C_R currents at picrotoxin concentrations between 0.5 µM and 50 µM (P<0.05). The approximately 6-fold difference in picrotoxin sensitivity suggests that homomeric ρ1 receptors may contribute more to the tonic GABA_C_R current than to synaptic GABA_C_R currents.

### GABA_C_R subunit composition: Cyclothiazide-sensitivity

**Figure 5:**
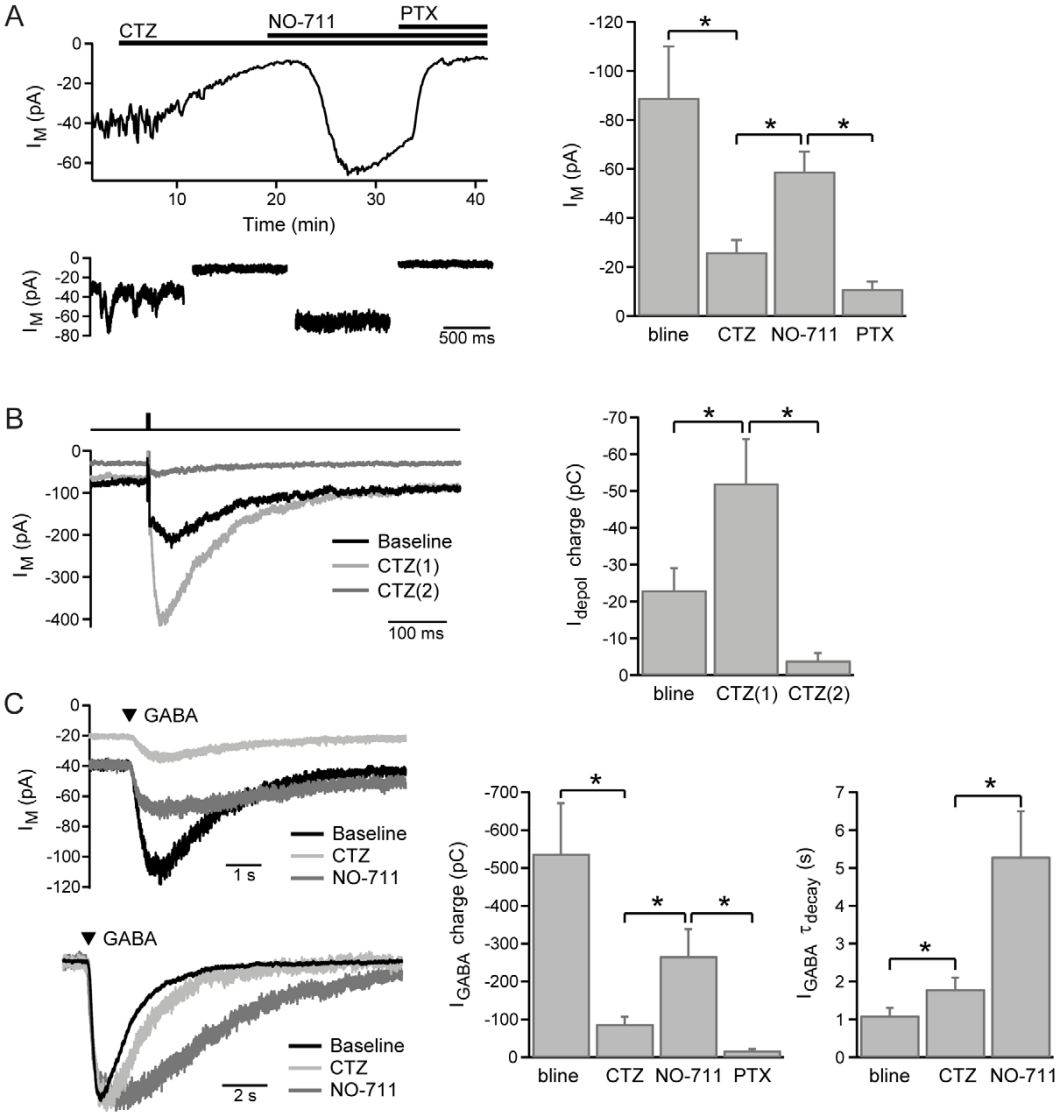
Cyclothiazide-sensitivity of GABA_C_R-currents. A, Example experiment showing the effect of cyclothiazide (CTZ; 300 µM) on the holding current, with subsequent application of NO-711 (3 µM) and picrotoxin (250 µM), with mean data for these experiments (n = 10). B, Example synaptic feedback currents evoked by brief BCT depolarization (to −10 mV for 5 ms) during wash-on of cyclothiazide, and mean data showing the biphasic effect of cyclothiazide on the charge of feedback responses (n = 8). CTZ(1) was measured 3–4 mins after application, CTZ(2) was measured 6–8 mins after application. C, Example GABA-evoked responses (100 µM, 50–100 ms) showing the effects of cyclothiazide and NO-711 on response amplitude (top) and kinetics (bottom, peak-scaled responses from a different experiment), with mean data (n = 7) for the charge and decay time-constant of GABA-evoked responses under these conditions.

Cyclothiazide has recently been shown to be a selective inhibitor of ρ2 receptors, acting as a non-competitive antagonist with an IC_50_ of ∼12 µM. At a concentration of 300 µM, cyclothiazide abolishes GABA responses mediated by ρ2 homomers but has no significant effect on the responses of ρ1 homomers [42]. We therefore examined the effect of cyclothiazide on GABA_C_R-mediated currents in BCTs. Bath-application of cyclothiazide (300 µM), in the presence of bicuculline (50 µM), significantly reduced the amplitude of the holding current (n = 10), and also reduced the spontaneous fluctuations of this current (fig. 5A). Synaptic feedback currents evoked by brief BCT depolarization (to −10 mV for 5 ms) were initially potentiated during cyclothiazide wash-on, as observed previously [43], due to the activity of cyclothiazide as an inhibitor of AMPA receptor desensitization. However, the feedback currents were subsequently virtually eliminated (n = 8; fig. 5B), although it is likely that run-down of BCT exocytosis contributed to the feedback current reduction [26]. GABA_C_R currents evoked by local application of GABA (100 µM, 50–100 ms) were also significantly reduced by cyclothiazide, but not completely eliminated (n = 7; fig. 5C). GABA-evoked responses had a slower rate of decay in the presence of cyclothiazide than in control conditions (n = 7; fig. 5C).

To further investigate the remaining cyclothiazide-resistant tonic and GABA-evoked currents, NO-711 (3 µM) was applied in the continuing presence of cyclothiazide. NO-711 increased the holding current (n = 10), which was subsequently inhibited by application of picrotoxin (200–250 µM; fig. 5A). In addition, NO-711 significantly increased the charge and slowed the decay of responses evoked by exogenous GABA (n = 7; fig. 5C). These results support the view that the majority of BCT GABA_C_Rs are ρ1-ρ2 heteromers, but provide evidence that a population of homomeric ρ1 receptors contributes to the tonic current.

## Discussion

The aim of the current experiments was to further our understanding of two unknown properties of the tonic GABA_C_R current in BCTs: the non-vesicular source of GABA for activating the current and the identity of the receptors mediating the current. Following recent reports of non-vesicular GABA release via Best1 anion channels [12], we tested the effects of anion channel inhibitors on the tonic GABA_C_R current. The results indicate that the GABA release mechanism is insensitive to NPPB and FFA but sensitive to DIDS. All three drugs inhibited to some extent the activity of GABA transporters, as evidenced by the potentiation of tonic, GABA-evoked and synaptic feedback currents mediated by GABA_C_Rs. In addition, NPPB and FFA exerted effects on intact BCs via inhibition of hemichannels, and DIDS was found to inhibit GABA_A_R-mediated sIPSCs. However, there appeared to be no direct inhibitory effect of NPPB, FFA or DIDS on GABA_C_Rs.

There is increasing evidence for the release of neurotransmitters, in particular glutamate and ATP, from astrocytes [44], [45]. GABA is also known to be released from astrocytes in the hippocampus, cerebellum, thalamus and olfactory bulb, with consequent activation of neuronal GABA_A_Rs [12], [46]-[48]. Astrocytes release ‘gliotransmitters’ via several mechanisms including Ca^2+^-dependent vesicular exocytosis, reversal of transporters, and release via hemichannels, ionotropic purinergic receptors and anion channels [49]. Various different types of anion channel have been implicated in gliotransmitter release including volume-regulated anion channels (VRACs) [15] and more recently Ca^2+^-activated anion channels such as Best1, which are present in hippocampal and cerebellar astrocytes, and which can mediate tonic GABA release [12], [13].

Distinguishing pharmacologically between mechanisms of non-vesicular release and between different types of Cl^-^ channel is challenging due to the cross-reactivity of commonly-used anion channel inhibitors with other release mechanisms, for example the block of hemichannels by NPPB [50], and due to the lack of selectivity of inhibitors between Cl^-^ channel classes [51]. However, the insensitivity of the tonic GABA_C_R current to carbenoxolone, PPADS and Brilliant Blue G [11], and to NPPB and FFA indicates that hemichannels, P2X_7_ receptors, VRACs and Best1 anion channels are not major contributors to the non-vesicular GABA release that activates this current. Reversal of GABA transporters also does not seem to be involved [11]. The non-vesicular release of GABA in the cerebellum that activates a tonic GABA_A_R current in granule cells was similarly found to be independent of GABA transporter reversal and VRACs, and to be potentiated rather than inhibited by NPPB [8].

The tonic GABA_C_R current in BCTs was significantly inhibited by DIDS, but the identity of the DIDS-sensitive anion channel or exchanger that mediates tonic GABA release in the retina is not known. Interestingly, a similar NPPB-resistant but DIDS-sensitive mechanism underlies the tonic release of glutamate in the hippocampus [33]. One potential candidate is a type a large-conductance Cl^-^ channel (maxi-Cl^-^) that was identified in drosophila and has three mammalian homologs that are activated by either Ca^2+^ or cell swelling, which is sensitive to DIDS but resistant to niflumic acid [52]. In the current experiments, DIDS failed to completely block the tonic GABA_C_R current in BCTs, even in Ca^2+^-free extracellular solution, suggesting that either DIDS at this concentration does not completely block the non-vesicular release mechanism, or it blocks only one of two or more contributing mechanisms. Alternatively, in the presence of DIDS the release of GABA may be blocked but, due to the additional action of DIDS as an inhibitor of GABA uptake, the ambient extracellular GABA concentration remains sufficient to evoke some tonic GABA_C_R current.

The cellular source of GABA for activating the tonic GABA_C_R current in BCTs is also unknown, but the most likely sources are amacrine cells and Müller cells. BCTs are surrounded by amacrine cell processes that make the conventional GABAergic synapses that mediate reciprocal and lateral feedback inhibition [53]. Although non-vesicular neurotransmitter release is thought to occur primarily from glial cells, DIDS-sensitive GABA-permeable anion channels have been observed in Deiters neurons in the brainstem [54]. Müller cells, the principle glial cells of the retina, are known to release neuroactive substances such as ATP, with consequent effects on synaptic activity and spiking in ganglion cells [55].

The tonic activation of membrane conductances as a result of non-vesicular neurotransmitter release may be a general feature of neuronal function in the CNS, involving not only inhibitory but also excitatory receptor systems. For example, the non-vesicular release of glutamate from astrocytes evokes a tonic NMDA receptor current in hippocampal neurons [33], [56]–[58]. A common feature of GABAergic and glutamatergic tonic currents is their potentiation by inhibition of neurotransmitter uptake, which may provide an endogenous regulatory system for controlling the magnitude of the current and its consequent effects on neuronal excitability [1].

BCs express both ρ1 and ρ2 GABA_C_R subunits, which readily form heteromeric receptors, and it is likely that most BC GABA_C_Rs are ρ1-ρ2 heteromers [22], [23], [59]-[61]. However, the additional expression of homomeric receptors would extend the functional diversity of GABA_C_R-mediated inhibition, as receptor properties are dependent on ρ subunit composition. Each ρ subunit has a similar structure to other members of the Cys-loop superfamily of ligand-gated ion channels [62]. Amino-acid substitutions in the pore-forming second transmembrane domain, in particular a switch at the 2′ position from proline in ρ1 to serine in ρ2, underlies subunit differences in properties such as deactivation rate, channel conductance and sensitivity to GABA [24], [63], [64]. This amino-acid substitution also underlies the difference in sensitivity to both picrotoxin and cyclothiazide of ρ1 and ρ2 receptors [41], [42].

We initially investigated the subunit composition of GABA_C_Rs in BCTs by comparing the picrotoxin-sensitivity of glu-evoked and tonic GABA_C_R currents. Glu-evoked currents, designed to predominantly activate synaptic GABA_C_Rs, were more sensitive to picrotoxin than the tonic GABA_C_R current, with IC_50_ values of 1.4 µM and 8.5 µM respectively. When expressed in heterologous systems, perch ρ1A and ρ1B homomeric receptors have reported IC_50_ values for picrotoxin inhibition of 10 µM and 56 µM, compared with 2 µM for ρ2A and ρ2B homomers [21], [23]. Heteromeric ρ1B/ρ2A receptors exhibit a similar sensitivity to ρ2 homomers when the ρ subunits are expressed at a 1∶1 ratio (IC_50_ value of 3 µM) [23]. A similar difference has been reported for human ρ subunits (eg. IC_50_ values of 48 µM for ρ1 and 5 µM for ρ2 homomeric receptors), with heteromeric receptors having an intermediate sensitivity [22], [41]. The subunit-specific differences in picrotoxin sensitivity are most pronounced in the presence of relatively high GABA concentrations (10–30 µM), due to a competitive component in the inhibition of ρ1 receptors [41]. The difference in picrotoxin sensitivity of the tonic and synaptic GABA_C_R currents in BCTs suggests that these currents may be mediated by different (though probably overlapping) populations of GABA_C_Rs, with a greater contribution of ρ1 receptors to the tonic current.

In addition we investigated the effect of cyclothiazide, which has recently been shown to be a selective inhibitor of ρ2 subunits [42]. Cyclothiazide reduced the amplitude of the tonic current, inhibited synaptic feedback currents and reduced the size of GABA-evoked responses, consistent with most BCT GABA_C_Rs being ρ1-ρ2 heteromers. The reduction in the amplitude and spontaneous fluctuations of the tonic current by cyclothiazide is similar to that observed with application of Ca^2+^-free solution [11], suggesting that the summation of slow IPSCs evoked by spontaneous synaptic release activating heteromeric GABA_C_Rs contributes to the tonic current in BCTs [5]. Spontaneous GABA release occurs at a high rate at amacrine cell to BCT synapses in retinal slices, as evidenced by the high frequency of GABA_A_R-mediated sIPSCs observed in the absence of bicuculline [11] (fig. 3). In the presence of bicuculline, synaptic GABA release and the tonic GABA_C_R current tend to be potentiated due to amacrine cell disinhibition [6].

However, a small constant tonic current remained in the presence of 300 µM cyclothiazide that was potentiated by inhibition of GABA uptake and is likely to be mediated by homomeric ρ1 receptors [42]. Small GABA-evoked currents were also observed in the presence of cyclothiazide that were potentiated by NO-711 and inhibited by picrotoxin. The slower decay rate of GABA-evoked currents in cyclothiazide compared with control conditions is consistent with reports of subunit-specific kinetics. For example, the deactivation rate of homomeric ρ1 receptors is slower than for ρ1-ρ2 heteromers, with respective time-constants of 14 s and 9 s for human subunits, and 234 s and 75 s for perch subunits (B form) [22], [23]. The change in decay kinetics also provides evidence against an incomplete block of heteromeric GABA_C_Rs by cyclothiazide. The lack of desensitization of BCT GABA_C_Rs [4] and the slow deactivation of ρ1 subunits are both likely to contribute to the very slow decay rate of GABA-evoked responses in the absence of GABA uptake (fig. 5). These properties, combined with a high affinity for GABA [21], [22], make homomeric ρ1 receptors particularly suitable for mediating a tonic current in BCTs.

Given the lack of dependence on vesicular release of the tonic GABA_C_R current [11], it is likely that the population of homomeric ρ1 receptors that contributes to this current is located extrasynaptically. An analogous situation is found in central neurons, where tonic GABA_A_R currents are mediated by extrasynaptic receptors [16]. Fluorescence imaging of immunolabeled ρ subunits in BCTs has shown ‘punctate’ labeling in several species including goldfish, with labeling within the synaptic cleft at the electron microscope level [65]–[68], reflecting the synaptic localization of heteromeric receptors that mediate GABA_C_R feedback currents and spontaneous IPSCs. However, it has been noted that rat BCTs also exhibit diffuse extrasynaptic ρ subunit labeling [68], which may correspond with a population of homomeric ρ1 receptors that contributes to the tonic current. Identification of the subcellular localization of GABA_C_R subunits in BCTs, and mechanisms that target specific receptors to synaptic or extrasynaptic sites, requires further investigation. In addition, it will be interesting to determine whether synaptic and extrasynaptic GABA_C_Rs are differentially regulated, and the relative importance of factors such as changes in receptor number or properties, or in the rates of GABA release and uptake, in modulating synaptic and tonic forms of GABA_C_R-mediated inhibition in BCTs.

In summary, these experiments indicate that tonic GABA_C_R currents in BCTs are activated by GABA released, in part, via a DIDS-sensitive mechanism, and that homomeric ρ1 receptors contribute to this current. Tonic inhibition regulates the ability of BCTs to fire Ca^2+^-dependent action potentials [4], and is likely to modulate the transmission of light responses to ganglion cells. However, how this form of inhibition interacts with synaptic GABA_A_R and GABA_C_R-mediated inhibition, and with the multiple additional forms of synaptic feedback that exist in BCTs, in the processing of visual information in the retina remains to be determined.
